# O026: Countrywide prevalence study of healthcare-associated infections in brazilian hospitals: preliminary results

**DOI:** 10.1186/2047-2994-2-S1-O26

**Published:** 2013-06-20

**Authors:** CMCB Fortaleza, MC Padoveze, C Kiffer, AL Barth, ICRS Carneiro, JLN Rodrigues, L Santos Filho, MJG Mello, MD Asensi, PP Gontijo Filho, MS Pereira, M Rocha, RS Kuchenbecker, ES Medeiros, ACC Pignatari

**Affiliations:** 1 Tropical Diseases, Faculdade de Medicina de Botucatu - UNESP - Univ Estadual Paulista, Botucatu, Brazil; 2 Public Health, Escola de Enfermagem - USP - Universidade de São Paulo, Brazil; 3 Laboratório Especial de Microbiologia Clínca, Universidade Federal de São Paulo, São Paulo, Brazil; 4 Universidade Federal do Rio Grande do Sul, Porto Alegre, Brazil; 5 UFPA - Universidade Federal do Pará, Belém, Brazil; 6 UFC - Universidade Federal do Ceará, Fortaleza, Brazil; 7 UFPB - Universidade Federal da Paraíba, João Pessoa, Brazil; 8 IMIP - Pernambuco, Recife, Brazil; 9 FIOCRUZ - Rio de Janeiro, Rio de Janeiro, Brazil; 10 Microbiology, UFU - Universidade Federal de Uberlandia, Uberlandia, Brazil; 11 UFGO - Universidade Federal de Goiás, Goiânia, Brazil; 12 UFRGS - Universidade Federal do Rio Grande do Sul, Porto Alegre, Brazil; 13 UNIFESP - Escola Paulista de Medicina, São Paulo, Brazil

## Introduction

The knowledge of burden of Healthcare-Associated Infections (HAI) in hospitals is essential to drive governmental strategies for its prevention and control.

## Objectives

To identify the prevalence of HAI in a representative sample of Brazilian hospitals.

## Methods

A team of trained nurses carried out a hospital-wide HAI point prevalence survey in 2012. A sample of hospitals from five Brazilian regions was evaluated (n=91; total of 8,853 beds).

## Results

The overall infection rate was 11.1%, varying from 2.5% (hospitals with <50 beds) to 18.3% (hospitals with > 200 beds). Reference hospitals showed 11.2% of overall infection rate. The most prevalent infections were pneumonia (3.6%), bloodstream infection (3.5%), surgical site infection (1.4%), urinary tract infection (1.1%) and skin infection (0.4%). Hospitals with >200 beds were likely to have higher HAI rates (RR=1.71; IC=1.398-2.10; P<0.001). The risk factors more frequently identified were: central venous catheter (17.8%), surgery (15.5%), urinary catheter (14.0%), and mechanical ventilators (8.1%). Etiologic agents were identified only in 9.1% (43/473) of infections. Gram-negative organisms were more frequent (56.0%), among them, *Klebsiella spp* (19.0%) and *Pseudomonas aeruginosa* (16%) and were predominant. Among Gram-positives (35.0%), coagulase-negative *Staphylococci* were more prevalent (16%) than *Staphylococcus aureus* (9.0%) or Enteroccoccus spp (6%). Yeasts were identified in 9.0% of HAI.

## Conclusion

These preliminary results emphasize both the relevance and the heterogeneity of HAI in Brazilian hospitals.

## Disclosure of interest

None declared.

